# *In vitro* cytotoxicity and virucidal efficacy of potassium hydrogen peroxymonosulfate compared to quaternary ammonium compound under various concentrations, exposure times and temperatures against African swine fever virus

**DOI:** 10.14202/vetworld.2021.2936-2940

**Published:** 2021-11-21

**Authors:** Watcharee Sovijit, Machimaporn Taesuji, Khate Rattanamas, Darsaniya Punyadarsaniya, Thanongsak Mamom, Hoa Thi Nguyen, Sakchai Ruenphet

**Affiliations:** 1 Department of Immunology and Virology, Faculty of Veterinary Medicine, Mahanakorn University of Technology, Bangkok, Thailand; 2 Department of Pathology, Faculty of Veterinary Medicine, Mahanakorn University of Technology, Bangkok, Thailand; 3 Key Laboratory of Veterinary Biotechnology, Faculty of Veterinary Medicine, Vietnam National University of Agriculture, Hanoi, Vietnam

**Keywords:** African swine fever, disinfectant, porcine alveolar macrophage cell, potassium hydrogen peroxymonosulfate, quaternary ammonium compound, virucidal efficacy

## Abstract

**Background and Aim:**

The selection and proper application of disinfectants are crucial to the prevention of many diseases, so disinfectants must be evaluated before being used for the prevention of African swine fever (ASF). Three disinfectant products belonging to the group of potassium hydrogen peroxymonosulfates, product A and product B, and a quaternary ammonium compound called product C, were examined *in vitro* for host cell cytotoxicity and the efficacy of ASF virus inactivation. The study parameters included various concentrations, exposure times, temperatures, and degrees of cytotoxicity.

**Materials and Methods:**

Three disinfectant products were evaluated for cytotoxicity using primary porcine alveolar macrophage (PAM) cells at dilutions from 1:200 to 1:51,200. Disinfectants in concentrations of 1:200, 1:400, and 1:800 were prepared, the pH and the virucidal activity were tested. An equal volume of each dilution was mixed with the ASF virus and incubated at room temperature (20°C) or on ice (4°C) for 1 min, 5 min, or 30 min. Hemadsorption (HAD) or rosette formation was observed using an inverted microscope for 5 days after inoculation, and the virus titer was calculated as HAD_50_/mL. Each treatment and virus control were tested in triplicate, and the titers were reported as means and standard deviations. The reduction factor was used to measure inactivation.

**Results:**

Products A, B, and C at 1:400, 1:800, and 1:25,600 of dilution, respectively, did not show significant cytotoxic effects on PAM cells. Products A and B could inactivate ASF virus at 1:200 dilution within 5 min after exposure at 4°C. However, at 20°C, the exposure time had to be extended to 30 min to inactivate the virus. Product C could inactivate the virus at 1:400 dilution within 5 min under both temperature conditions, whereas at 1:800 dilution, the exposure time had to be extended to 30 min to completely inactivate the virus at 20°C.

**Conclusion:**

All disinfectants could inactivate ASF virus in various concentrations, under appropriate exposure times and reaction temperatures, and there was no evidence of host cell cytotoxicity. For the control of ASF in pig farms, the appropriate concentration, ambient temperature, and contact time of these disinfectants should be taken into account.

## Introduction

African swine fever virus (ASFV) is the causative agent of ASF in pigs. The disease was first reported in east Africa in the early 1900s. At present, ASFV is on the World Organization for Animal Health list of notifiable diseases, due to its high morbidity and mortality, and substantial economic losses [1-4]. ASFV is classified in the family *Asfarviridae*, genus *Asfivirus*. It is a large enveloped DNA virus, with an icosahedral capsid structure [5,6]. ASFV can persist in the environment around pig farms, in carcasses and swine products as well as in various fomites such as pig houses, transport cars and tracks, and slaughterhouses. However, several studies have reported that ASFV can infect commercial pigs through vectors such as warthogs (*Phacochoerus africanus*), bush pigs (*Potamochoerus porcus* and *Potamochoerus larvatus*), and soft ticks (*Ornithodoros moubata*) [4], to which the virus is transmitted by trans-stadial and transovarial routes [7].

The prevention of ASFV is important to reduce the risk of infection in commercial pig farms, and strategies for cleaning and disinfection on and around pig farms are important [8]. ASFV may remain and cause infection for months to years in feces and blood. However, in the presence of organic materials and contamination on various surfaces, ASFV might be more stable and may even survive longer, especially in waste products around pig houses [9,10]. Hence, the choice of a suitable disinfectant and its effective application is important. Environmental conditions, contact time, pH, and temperature ranges play crucial roles in controlling ASFV.

In the present study, three commercial disinfectant products belonging to the groups of potassium hydrogen peroxymonosulfate and quaternary ammonium compounds were evaluated for their virucidal efficacy against ASFV and host cell cytotoxicity, using primary porcine alveolar macrophage (PAM) cells under various concentrations, exposure times, and temperatures.

## Materials and Methods

### Ethical approval

In the present study, we euthanized healthy piglets to collect the lung preparing primary PAMs culture for *in vitro* study. This study was approved by the Animal Welfare and Ethics Committee of Vietnam National University of Agriculture, Vietnam, and the ethics approval number is VNUA-2021/05.

### Study period and location

This study was conducted from March 2021 to June 2021. The pigs were housed and used in an isolated area in the Biosecurity Animal Facility Centre of the Vietnam National University of Agriculture (VNUA), Hanoi, Vietnam.

### Cell preparation

The PAM cells were prepared from 7-week-old healthy piglets which were not identified by polymerase chain reaction as having ASFV, porcine circovirus, classical swine fever virus, or porcine respiratory and reproductive syndrome virus, and were seronegative for ASFV. Primary PAM cells were cultured in growth medium containing an RPMI 1640 medium (Gibco, Thermo Fisher Scientific, Waltham, MA, USA), 10% fetal calf serum (FCS; Gibco), and 1% penicillin-streptomycin solution (Gibco). The cells were cultured and incubated at 37°C in a 5% CO_2_ incubator. Pig red blood cells (Percoll, GE Healthcare, Chicago, IL, USA) were prepared and mixed in maintenance medium (MM) which contained RPMI 1640 medium and 1% penicillin-streptomycin solution, and were kept at 4°C before testing.

### Disinfectants

Three disinfectant products belonging to the group of potassium hydrogen peroxymonosulfates, product A and product B, and a quaternary ammonium compound called product C, were examined *in vitro* for host cell cytotoxicity and the efficacy of ASFV inactivation. All disinfectants were manufactured by Mixwell Marketing Co., Ltd., Bangkok, Thailand. Each product was diluted with distilled water as 1:200, 1:400, and 1:800 up to 1:51,200, prior to testing.

### Virus preparation

The ASFV, VNUA-ASFV-L01/HN/04/19, was propagated in PAM cells following the method published by Truong *et al*. [11]. After harvesting, ASFV samples were aliquoted and kept at −80°C until testing. In the present study, the viral concentration was used at least 6.5 log10 hemadsorption (HAD)_50_/mL. Viral propagation and preparation were performed in a biohazard cabinet Class II of Key Laboratory of Veterinary Biotechnology, Faculty of Veterinary Medicine, Vietnam National University of Agriculture, Hanoi, Vietnam.

### Cytotoxicity testing

All disinfectants were evaluated for *in vitro* cytotoxicity using PAM cells at 1:200 to 1:51,200 disinfectant dilution. Briefly, each diluted disinfectant was added to PAM cells, which were incubated for 1 h, before MM with 2% pig red blood cells was added and incubated at 37°C in a 5% CO_2_ incubator. The number of viable cells was determined under an inverted microscope (Motic, Fujian, China). The cutoff of cell viability was determined when live cells clung to the bottom of the tissue culture microplate for more than 80% of the 5 days culture period, equivalent to the cell control well.

### Virucidal efficacy

Dilutions of each disinfectant product of 1:200, 1:400, and 1:800 were prepared before pH checking and virucidal testing. An equal volume of each dilution was mixed with ASFV, then incubated at room temperature (20°C) or on ice (4°C) for 1, 5, or 30 min. The mixture was then diluted 10-fold using MM and inoculated onto PAM cells for virus recovery. These treatments were incubated in a 5% CO_2_ incubator and observed twice a day for 5 days. The HAD or rosette formation was observed using an inverted microscope for 5 days. The virus titer was calculated as HAD_50_/mL, following the Reed and Muench [12] and Taesuji *et al*. [13] methods. Each treatment and virus control were tested in triplicate, and the titers were reported as means and standard deviations.

### Inactivation analysis

The reduction factor (RF) was used to measure inactivation. The RF was calculated as follows: RF=t_pc_−t_a_, where t_pc_ is the titer converted into an index in log_10_ of the virus control and t_a_ is the titer converted into index in log_10_ of the recovered virus from the treated sample. ASFV inactivation was considered to be effective when the RF was greater than or equal to 3 [14-16].

### Results

The cytotoxicity of various concentrations of three commercially disinfectants was assessed using primary PAM and pig red blood cells (Table-1). Products A, B, and C did not show any significant effect on cells at dilutions of 1:400, 1:800, or 1:25,600, respectively.

**Table-1:** The percentage of alive primary PAM cells after incubation with a certain disinfectant of various concentrations.

Dilutions	Percentage of PAM cell viability			

	Cell control	Product A	Product B	Product C
1:200	100	40	0	0
1:400	100	90	0	0
1:800	100	100	90	0
1:1600	100	100	100	0
1:3200	100	100	100	0
1:6400	100	100	100	0
1:12,800	100	100	100	70
1:25,600	100	100	100	100
1:51,200	100	100	100	100

Cutoff of cells viability was indicated when live cells clung to the bottom of tissue culture microplate more than 80% of the 5-day culturing period. PAM=Porcine alveolar macrophage

The pH of product A at dilutions of 1:200, 1:400, and 1:800 was 2.83, 3.57, and 6.16, respectively. The pH of product B was 3.21, 4.46, and 6.38, and product C was 7.64, 7.57, and 7.53, respectively.

The inactivation of ASFV by three commercial disinfectants under various concentrations, two different temperatures, and different exposure times are shown in Table-2. Products A and B could inactivate ASFV at dilutions of 1:200 within five minutes of exposure at 4°C. However, at 20°C, an extension of exposure time to 30 min was required for viral inactivation. Product C could inactivate the virus at a dilution of 1:400 within 5 min under both temperatures, whereas an exposure time of up to 30 min was required when using a 1:800 dilution, to inactivate the virus at 20°C.

**Table-2:** Virucidal efficacy of two potassium hydrogen peroxymonosulfate disinfectants and a quaternary ammonium compound disinfectant against African swine fever virus under various concentrations, exposure times, and temperatures.

Product	Temperature (°C)	Exposure time (min)	Virus control (log10 HAD_50_/mL)	Mean±SD of titer reduction (log10 HAD_50_/mL)		

				1:200	1:400	1:800
A	4	1	6.82±0.15	2.62±0.41	1.97±0.16	0.75±0.28
		5	6.84±0.08	3.01±0.18*	2.04±0.19	0.88±0.21
		30	6.86±0.10	3.83±0.16*	2.39±0.37	1.16±0.03
	20	1	6.95±0.03	2.42±0.47	1.87±0.08	0.64±0.15
		5	6.80±0.00	2.61±0.35	1.94±0.10	0.82±0.00
		30	6.96±0.03	4.05±0.22*	2.50±0.36	1.30±0.01
B	4	1	6.76±0.07	2.79±0.11	2.41±0.36	1.74±0.36
		5	6.84±0.08	3.12±0.13*	2.65±0.46	1.94±0.28
		30	6.86±0.10	4.32±0.02*	3.11±0.36*	1.97±0.27
	20	1	6.83±0.18	2.71±0.36	2.18±0.35	1.62±0.43
		5	6.76±0.07	2.94±0.10	2.29±0.51	1.68±0.30
		30	6.88±0.17	4.58±0.27*	2.90±0.67	2.04±0.18
C	4	1	6.85±0.14	≥4.05±0.14**	3.17±0.40	2.24±0.02
		5	6.88±0.17	≥4.08±0.17**	3.63±0.50	2.45±0.13
		30	6.86±0.10	≥4.06±0.10**	≥4.06±0.10**	2.90±0.13
	20	1	6.70±0.09	≥3.90±0.09**	3.23±0.66*	2.64±0.20
		5	6.66±0.03	≥3.86±0.03**	3.64±0.37*	2.85±0.31
		30	6.80±0.13	≥4.00±0.13**	3.96±0.07*	3.32±0.29*

* Virus inactivation was regarded as effective when reduction factor was≥3.

** Indicates that recovery virus could not be detected in that condition, so the reduction factor was showed≥mean±SD and also significantly inactivating effective. HAD=Hemadsorption, SD=Standard deviation

## Discussion

In the present study, primary PAM cells were used for the testing of cytotoxicity and virucidal efficacy. The metric used to identify unaffected cells was the presence of 80% of alive cells or clinging to the bottom of the tissue culture microplate. We evaluated virucidal efficacy using the cytopathic effect on infected cells demonstrated by pig red blood cell adsorption around PAM cells, also called HAD or rosette formation [17-19]. Several researchers have used this characteristic to indicate the presence of ASFV in the tissue culture [10,11].

Three of the chosen commercial disinfectants were examined for cytotoxicity and ASFV inactivation. Products A and B were based on potassium hydrogen peroxymonosulfate, also known as potassium peroxymonosulfate, and both products contained potassium hydrogen peroxymonosulfate as active oxygen 2.5% w/w and sodium lauryl sulfate 2.9% w/w. Product C was based on a quaternary ammonium compound which consisted of several active ingredients including alkyl dimethyl benzyl ammonium chloride 2.20% w/v, octyl decyl dimethyl ammonium chloride 1.65% w/v, dioctyl dimethyl ammonium chloride 0.66% w/v, didecyl dimethyl ammonium chloride 0.99% w/v polyethoxylated propoxylated alkyl alcohol 2.50% w/v, and sodium metasilicate 0.50% w/v. The results of this study indicated that the virucidal efficacy of product C against ASFV was greater than that of product B and product A under the same *in vitro* test conditions. However, the degree of *in vitro* cytotoxicity to PAM cells of product A was less than that of product B and product C. These results implied that product A might cause less host cell damage than product B and product C under the same conditions. Several researchers have reported that oxidizing agents, especially potassium salt, are widely used for the inactivation of bacteria and viruses, and their virucidal efficacy was better than that of the quaternary ammonium compound [20,21]. However, several publications, especially Juszkiewicz *et al*. [22] and Shirai *et al*. [23], indicated that quaternary ammonium compounds also inactivate enveloped viruses and ASFV at room temperature (20°C) within 30 min. Moreover, the quaternary ammonium compounds used in the present study consisted of several active ingredients, called “commercially supply compound disinfectants,” or “cocktail disinfectants,” hence, it is possible that product C enhanced the synergistic effect of each main agent/ingredient, which might increase its virucidal efficacy. This could explain why ASFV inactivation using quaternary ammonium compound was better than that using potassium hydrogen peroxymonosulfate.

## Conclusion

All of the disinfectants used in this study were able to inactivate ASFV *in vitro* under various concentrations, exposure times, and temperatures. All products could be used as disinfectants, especially for biosecurity enhancement aiming to control ASF in pig farms. To obtain the highest efficacy, users have to select appropriate concentrations and ensure a sufficient contact time for a given environmental climate.

## Authors’ Contributions

WS, MT, KR, DP, TM, HTN, and SR: Contributed to the study conception, design, conducted the experiments, and analyzed the data. SR: Contributed to sample preparation. TM and SR: Drafted the manuscript. All authors read and approved the final manuscript.
